# The genome sequence of *Sphagnum tenellum* (Brid.) Bory, 1819 (Sphagnales: Sphagnaceae)

**DOI:** 10.12688/wellcomeopenres.26663.1

**Published:** 2026-05-29

**Authors:** David Bell, David G. Long, Elizabeth M. Kungu, Royal Botanic Garden Edinburgh Genome Acquisition Lab, Plant Genome Sizing Collective, Darwin Tree of Life Barcoding collective, Wellcome Sanger Institute Tree of Life Management, Samples and Laboratory team, Wellcome Sanger Institute Scientific Operations: Sequencing Operations, Wellcome Sanger Institute Tree of Life Core Informatics team, Tree of Life Core Informatics collective, Darwin Tree of Life Consortium

**Affiliations:** 1 Royal Botanic Garden Edinburgh, Edinburgh, Scotland, UK

**Keywords:** Sphagnum tenellum, soft bog-moss, genome sequence, chromosomal, Sphagnales

## Abstract

We present a genome assembly of *Sphagnum tenellum* (soft bog-moss; Streptophyta; Sphagnopsida; Sphagnales; Sphagnaceae). The genome sequence has a total length of 386.16 megabases. Most of the assembly (98.04%) is scaffolded into 21 chromosomal pseudomolecules. The mitochondrial sequence has a length of 141.31 kilobases and the plastid genome assembly has a length of 140.16 kilobases. This assembly was generated as part of the Darwin Tree of Life project, which produces reference genomes for eukaryotic species found in Britain and Ireland.

## Species taxonomy

Eukaryota; Viridiplantae; Streptophyta; Sphagnopsida; Sphagnales; Sphagnaceae; *Sphagnum*; *Sphagnum tenellum* (Brid.) Bory, 1819 (NCBI:txid128247).

## Background

*Sphagnum tenellum* (Brid.) Bory, also known as soft bogmoss, is a small and delicate species of peat moss. It is a widespread member of the Suboceanic Boreo-temperate floristic element and common in north and west Britain and Ireland, but has declined significantly in the lowlands since 2000 ( Blockeel *et al.*, 2014). *Sphagnum tenellum* grows in loose tussocks or as scattered stems in wet, acidic habitats such as bogs and heaths, and also on heathy banks, crags and sheltered crevices among rocks ( Blockeel *et al.*, 2014).

*Sphagnum tenellum* is a dioicous species, producing male and female reproductive structures on separate plants. Capsules are frequent in Britain and Ireland, maturing earlier in the summer than those of other Sphagnum species ( Blockeel *et al.*, 2014).

The genome of *Sphagnum tenellum* was sequenced as part of the Darwin Tree of Life Project, a collaborative effort to sequence all named eukaryotic species in Britain and Ireland. The chromosomally complete genome presented here is consistent with the genome size estimate reported by Temsch *et al*. (1998) and the chromosome count reported by Smith & Newton (1968).

We anticipate this high-quality reference genome will be a valuable genomic resource for a range of future studies in this important plant group. Another chromosome-level assembly for this species is also available (GCA_965154135.1) submitted by Elixir Norway (data obtained via NCBI datasets, O’Leary *et al.*, 2024).

## Methods

### Sample acquisition, flow cytometry and DNA barcoding

**Figure 1.**
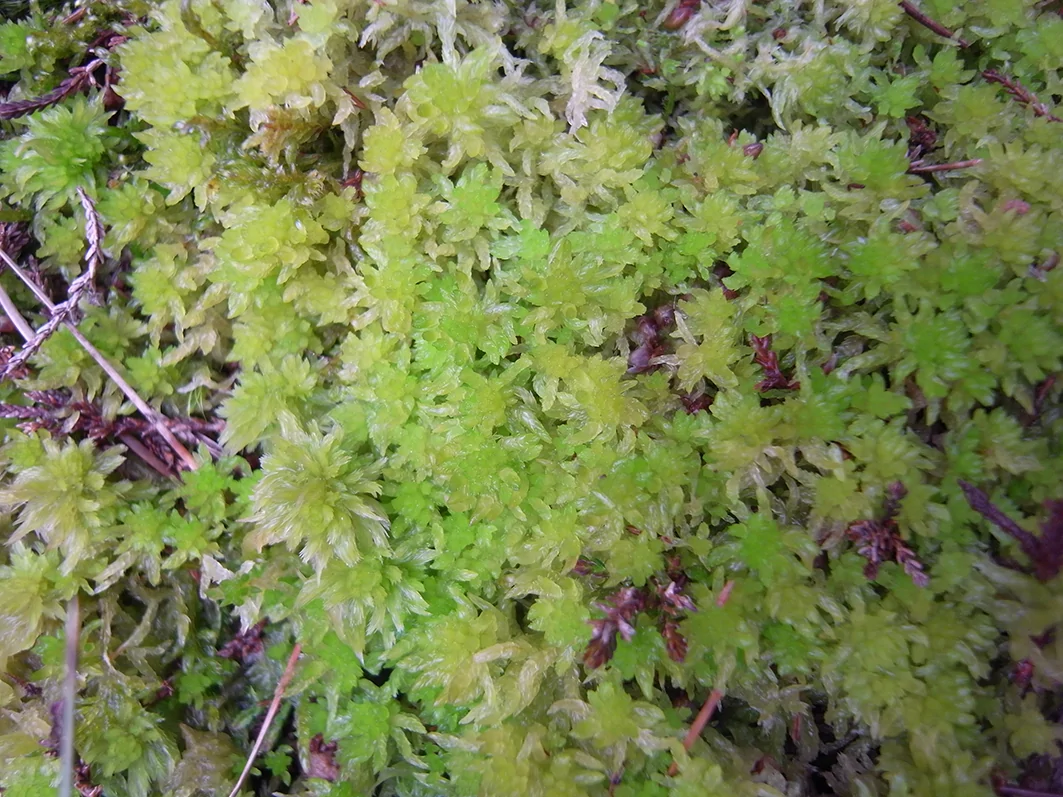
Photograph of the *Sphagnum tenellum* (csSphTene1) population from which samples were taken for genome sequencing.

A specimen of *Sphagnum tenellum* (specimen ID EDTOL01633, ToLID csSphTene1; Figure 1) was used for genome sequencing. It was collected from Crunklie Moss, Talla & Gameshope Borders Forest Trust Reserve, Peeblesshire, Scotland, UK (latitude 55.439, longitude −3.3677) on 2021-05-26. Other specimens collected on the same occasion were used for Hi-C sequencing (specimen ID EDTOL01638, ToLID csSphTene6) and RNA sequencing (specimen ID EDTOL01633, ToLID csSphTene1). The specimens were collected and identified by David Bell, David Long and Liz Kungu. The herbarium specimen associated with the sequenced plant is kept at the Royal Botanic Garden Edinburgh (E) https://data.rbge.org.uk/herb/E01152102.

The genome size was estimated by flow cytometry following the ‘one-step’ method outlined in Pellicer *et al*. (2021) and using propidium iodide as the fluorochrome. CyStain PI OxProtect Staining Buffer (Sysmex UK Ltd) was used for isolation of nuclei ( Loureiro *et al.*, 2007), and the internal calibration standard was *Oryza sativa* ‘IR36’ with an assumed 1C-value of 493.89 Mb ( Obermayer *et al.*, 2002).

The initial identification was verified by an additional DNA barcoding process according to the framework developed by Twyford *et al*. (2024). Part of the plant specimen was preserved in silica gel desiccant ( Chase & Hills, 1991). DNA extracted from the dried plant was amplified by PCR for standard barcode markers, with the amplicons sequenced and compared to public sequence databases including GenBank and the Barcode of Life Database (BOLD) ( Ratnasingham & Hebert, 2007). Following whole genome sequence generation, the relevant DNA barcode region was also used alongside the initial barcoding data for sample tracking at the WSI ( Twyford *et al.*, 2024). The standard operating procedures for Darwin Tree of Life barcoding are available on protocols.io.

### Nucleic acid extraction

Protocols for high molecular weight (HMW) DNA extraction developed at the Wellcome Sanger Institute (WSI) Tree of Life Core Laboratory are available on protocols.io ( Howard *et al.*, 2025). The csSphTene1 sample was weighed and triaged to determine the appropriate extraction protocol. For HMW DNA extraction, 55 mg of shoot tissue was used. Tissue from the shoot was homogenised by cryogenic disruption using the Covaris cryoPREP ^^®^^ Automated Dry Pulverizer. HMW DNA was extracted using the Automated Plant MagAttract v2 protocol. We used centrifuge-mediated fragmentation to produce DNA fragments in the 8–10 kb range, following the Covaris g-TUBE protocol for ultra-low input (ULI). Sheared DNA was purified by manual SPRI (solid-phase reversible immobilisation), using (Pacific Biosciences) AMPure PB beads to eliminate shorter fragments and concentrate the DNA. The concentration of the sheared and purified DNA was assessed using a Nanodrop spectrophotometer and Qubit Fluorometer using the Qubit dsDNA High Sensitivity Assay kit. Fragment size distribution was evaluated by running the sample on the FemtoPulse system. For this sample, the final post-shearing DNA had a Qubit concentration of 3.3 ng/μL and a yield of 155.10 ng, with a fragment size of 8.4 kb.

RNA was extracted from shoot tissue of csSphTene1 in the Tree of Life Laboratory at the WSI using the RNA Extraction: Automated MagMax™ *mir*Vana protocol. The RNA concentration was assessed using a Nanodrop spectrophotometer and a Qubit Fluorometer using the Qubit RNA Broad-Range Assay kit. Analysis of the integrity of the RNA was done using the Agilent RNA 6000 Pico Kit and Eukaryotic Total RNA assay.

### PacBio HiFi library preparation and sequencing

Library preparation and sequencing were performed at the WSI Scientific Operations core. Prior to library preparation, the DNA was fragmented to ~10 kb. Ultra-low-input (ULI) libraries were prepared using the PacBio SMRTbell® Express Template Prep Kit 2.0 and gDNA Sample Amplification Kit. Samples were normalised to 20 ng DNA. Single-strand overhang removal, DNA damage repair, and end-repair/A-tailing were performed according to the manufacturer’s instructions, followed by adapter ligation. A 0.85× pre-PCR clean-up was carried out with Promega ProNex beads.

The DNA was evenly divided into two aliquots for dual PCR (reactions A and B), both following the manufacturer’s protocol. A 0.85× post-PCR clean-up was performed with ProNex beads. DNA concentration was measured using a Qubit Fluorometer v4.0 (Thermo Fisher Scientific) with the Qubit HS Assay Kit, and fragment size was assessed on an Agilent Femto Pulse Automated Pulsed Field CE Instrument (Agilent Technologies) using the gDNA 55 kb BAC analysis kit. PCR reactions A and B were then pooled, ensuring a total mass of ≥500 ng in 47.4 μl.

The pooled sample underwent another round of DNA damage repair, end-repair/A-tailing, and hairpin adapter ligation. A 1× clean-up was performed with ProNex beads, followed by DNA quantification using the Qubit and fragment size analysis using the Agilent Femto Pulse. Size selection was performed on the Sage Sciences PippinHT system, with target fragment size determined by Femto Pulse analysis (typically 4–9 kb). Size-selected libraries were cleaned with 1.0× ProNex beads and normalised to 2 nM before sequencing.

The sample was sequenced using the Sequel IIe system (Pacific Biosciences, California, USA). The concentration of the library loaded onto the Sequel IIe was in the range 40–135 pM. The SMRT link software, a PacBio web-based end-to-end workflow manager, was used to set-up and monitor the run, and to perform primary and secondary analysis of the data upon completion.

### Hi-C

***Sample preparation and crosslinking***

Hi-C data were generated from the shoot tissue of csSphTene6 using the Arima-HiC v2 kit (Arima Genomics). Tissue was finely ground using the Covaris cryoPREP Dry Pulverizer (Covaris), and then subjected to nuclei isolation. Nuclei were isolated using a modified protocol based on the Qiagen QProteome Cell Compartment Kit (Qiagen), in which only the Lysis and CE2 buffers were used, with QIAshredder spin columns. After isolation, nuclei were fixed using formaldehyde to a final concentration of 2% to crosslink the DNA. The crosslinked DNA was then digested and biotinylated according to the manufacturer’s instructions. A clean-up step was performed with SPRIselect beads before library preparation. DNA concentration was quantified using the Qubit Fluorometer v4.0 (Thermo Fisher Scientific) and the Qubit HS Assay Kit, following the manufacturer’s instructions.

***Hi-C library preparation and sequencing***

Biotinylated DNA constructs were fragmented using a Covaris E220 sonicator and size selected to 400–600 bp using SPRISelect beads. DNA was enriched with Arima-HiC v2 kit Enrichment beads. End repair, A-tailing, and adapter ligation were carried out with the NEBNext Ultra II DNA Library Prep Kit (New England Biolabs), following a modified protocol where library preparation occurs while DNA remains bound to the Enrichment beads. Library amplification was performed using KAPA HiFi HotStart mix and a custom Unique Dual Index (UDI) barcode set (Integrated DNA Technologies). Depending on sample concentration and biotinylation percentage determined at the crosslinking stage, libraries were amplified with 10–16 PCR cycles. Post-PCR clean-up was performed with SPRISelect beads. Libraries were quantified using the AccuClear Ultra High Sensitivity dsDNA Standards Assay Kit (Biotium) and a FLUOstar Omega plate reader (BMG Labtech).

Prior to sequencing, libraries were normalised to 10 ng/μL. Normalised libraries were quantified again to create equimolar and/or weighted 2.8 nM pools. Pool concentrations were checked using the Agilent 4200 TapeStation (Agilent) with High Sensitivity D500 reagents before sequencing. Sequencing was performed using paired-end 150 bp reads on the Illumina NovaSeq 6000.

### RNA library preparation and sequencing

Libraries were prepared using the NEBNext ^^®^^ Ultra™ II Directional RNA Library Prep Kit for Illumina (New England Biolabs), following the manufacturer’s instructions. Poly(A) mRNA in the total RNA solution was isolated using oligo (dT) beads, converted to cDNA, and uniquely indexed; 14 PCR cycles were performed. Libraries were size-selected to produce fragments between 100–300 bp. Libraries were quantified, normalised, pooled to a final concentration of 2.8 nM, and diluted to 150 pM for loading. Sequencing was carried out on the Illumina NovaSeq 6000 to generate 150-bp paired-end reads.

### Genome assembly

Prior to assembly of the PacBio HiFi reads, a database of *k*-mer counts ( *k* = 31) was generated from the filtered reads using FastK. GenomeScope2 ( Ranallo-Benavidez *et al.*, 2020) was used to analyse the *k*-mer frequency distributions, providing estimates of genome size, heterozygosity, and repeat content.

The HiFi reads were assembled using Hifiasm ( Cheng *et al.*, 2021) with the —primary and -l0 options. Internal Hifiasm purging was switched off with -l0. Haplotypic duplications were identified and removed using purge_dups ( Guan *et al.*, 2020). The Hi-C reads ( Rao *et al.*, 2014) were mapped to the primary contigs using bwa-mem2 ( Vasimuddin *et al.*, 2019), and the contigs were scaffolded in YaHS ( Zhou *et al.*, 2023) with the —break option for handling potential misassemblies. The scaffolded assemblies were evaluated using Gfastats ( Formenti *et al.*, 2022), BUSCO ( Manni *et al.*, 2021) and MerquryFK ( Rhie *et al.*, 2020).

The organelle genomes were assembled using MitoHiFi ( Uliano-Silva *et al.*, 2023).

### Assembly curation

The assembly was decontaminated using the Assembly Screen for Cobionts and Contaminants ( ASCC) pipeline. TreeVal was used to generate the flat files and maps for use in curation. Manual curation was conducted primarily in PretextView and HiGlass ( Kerpedjiev *et al.*, 2018). Scaffolds were visually inspected and corrected as described by Howe *et al*. (2021). Manual corrections included nine breaks and 55 joins. This reduced the scaffold count by 62.7%, increased the scaffold N50 by 7.0%, and reduced the total assembly length by 5.8%. The curation process is described at https://gitlab.com/wtsi-grit/rapid-curation . PretextSnapshot was used to generate a Hi-C contact map of the final assembly.

### Assembly quality assessment

The MerquryFK tool ( Rhie *et al.*, 2020) was run in a Singularity container ( Kurtzer *et al.*, 2017) to evaluate *k*-mer completeness and assembly quality for the haploid assembly using the *k*-mer database ( *k* = 31) computed prior to genome assembly. The analysis outputs included assembly QV scores and completeness statistics.

The genome was analysed using the BlobToolKit pipeline, a Nextflow implementation of the earlier Snakemake version ( Challis *et al.*, 2020). The pipeline aligns PacBio reads using minimap2 ( Li, 2018) and SAMtools ( Danecek *et al.*, 2021) to generate coverage tracks. It runs BUSCO ( Manni *et al.*, 2021) using lineages identified by querying NCBI datasets ( O’Leary *et al.*, 2024). For the three domain-level lineages, BUSCO genes are aligned to the UniProt Reference Proteomes database ( Bateman *et al.*, 2023) using DIAMOND blastp ( Buchfink *et al.*, 2021). The genome is divided into chunks based on the density of BUSCO genes from the closest taxonomic lineage, and each chunk is aligned to the UniProt Reference Proteomes database with DIAMOND blastx. Sequences without hits are chunked using seqtk and aligned to the NT database with blastn ( Altschul *et al.*, 1990). The BlobToolKit suite consolidates all outputs into a blobdir for visualisation. The BlobToolKit pipeline was developed using nf-core tooling ( Ewels *et al.*, 2020) and MultiQC ( Ewels *et al.*, 2016), with containerisation through Docker ( Merkel, 2014) and Singularity ( Kurtzer *et al.*, 2017).

## Genome sequence report

### Sequence data

**Figure 2.**
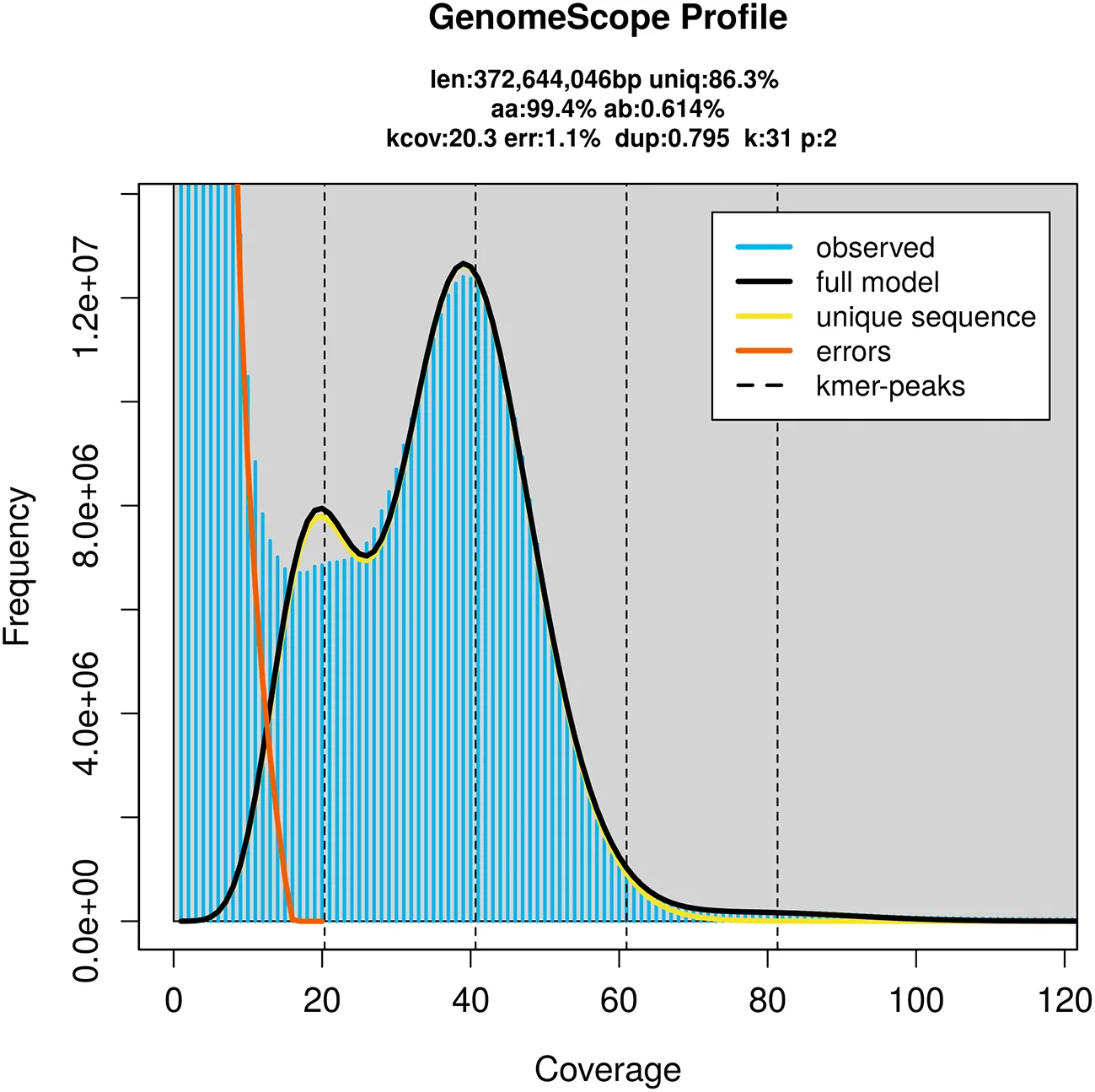
Frequency distribution of *k*-mers generated using GenomeScope2. The plot shows observed and modelled *k*-mer spectra, providing estimates of genome size, heterozygosity, and repeat content based on unassembled sequencing reads.

The genome of a specimen of *Sphagnum tenellum* was sequenced using Pacific Biosciences single-molecule HiFi long reads, generating 22.48 Gb (gigabases) from 2.31 million reads, which were used to assemble the genome. GenomeScope2.0 analysis estimated the haploid genome size at 372.97 Mb, with a heterozygosity of 0.61% and repeat content of 13.79% ( Figure 2). Using flow cytometry, the genome size (1C-value) of the sample was estimated to be 0.45 pg, equivalent to 440.00 Mb. These estimates guided expectations for the assembly. Based on the estimated genome size, the sequencing data provided approximately 41× coverage. Hi-C sequencing produced 130.06 Gb from 430.65 million reads, which were used to scaffold the assembly. RNA sequencing data were also generated and are available in public sequence repositories. Table 1 summarises the specimen and sequencing details.

**Table 1.** Specimen and sequencing data for *Sphagnum tenellum* (BioProject PRJEB65221).

Platform	PacBio HiFi	Hi-C	RNA-seq
**ToLID**	csSphTene1	csSphTene6	csSphTene1
**Specimen ID**	EDTOL01633	EDTOL01638	EDTOL01633
**BioSample (source individual)**	SAMEA10332304	SAMEA10332309	SAMEA10332304
**BioSample (tissue)**	SAMEA10332488	SAMEA10332493	SAMEA10332488
**Tissue**	shoot	shoot	shoot
**Instrument**	Sequel IIe	Illumina NovaSeq 6000	Illumina NovaSeq 6000
**Run accessions**	ERR11867221	ERR11872590	ERR11872591
**Read count total**	2.31 million	430.65 million	29.76 million
**Base count total**	22.48 Gb	130.06 Gb	8.99 Gb

### Assembly statistics

The haploid genome was assembled as a single assembly. The final assembly has a total length of 386.16 Mb in 558 scaffolds, with 639 gaps, and a scaffold N50 of 21.22 Mb ( Table 2).

**Table 2.** Genome assembly data for *Sphagnum tenellum.*

Genome assembly	Haploid assembly
**Assembly name**	csSphTene1.2
**Assembly accession**	GCA_963921925.2
**Assembly level**	chromosome
**Span (Mb)**	386.16
**Number of chromosomes**	21
**Number of contigs**	1 197
**Contig N50**	1.03 Mb
**Number of scaffolds**	558
**Scaffold N50**	21.22 Mb
**Organelles**	Mitochondrial genome: 141.31 kb; Plastid genome: 140.16 kb

**Figure 3.**
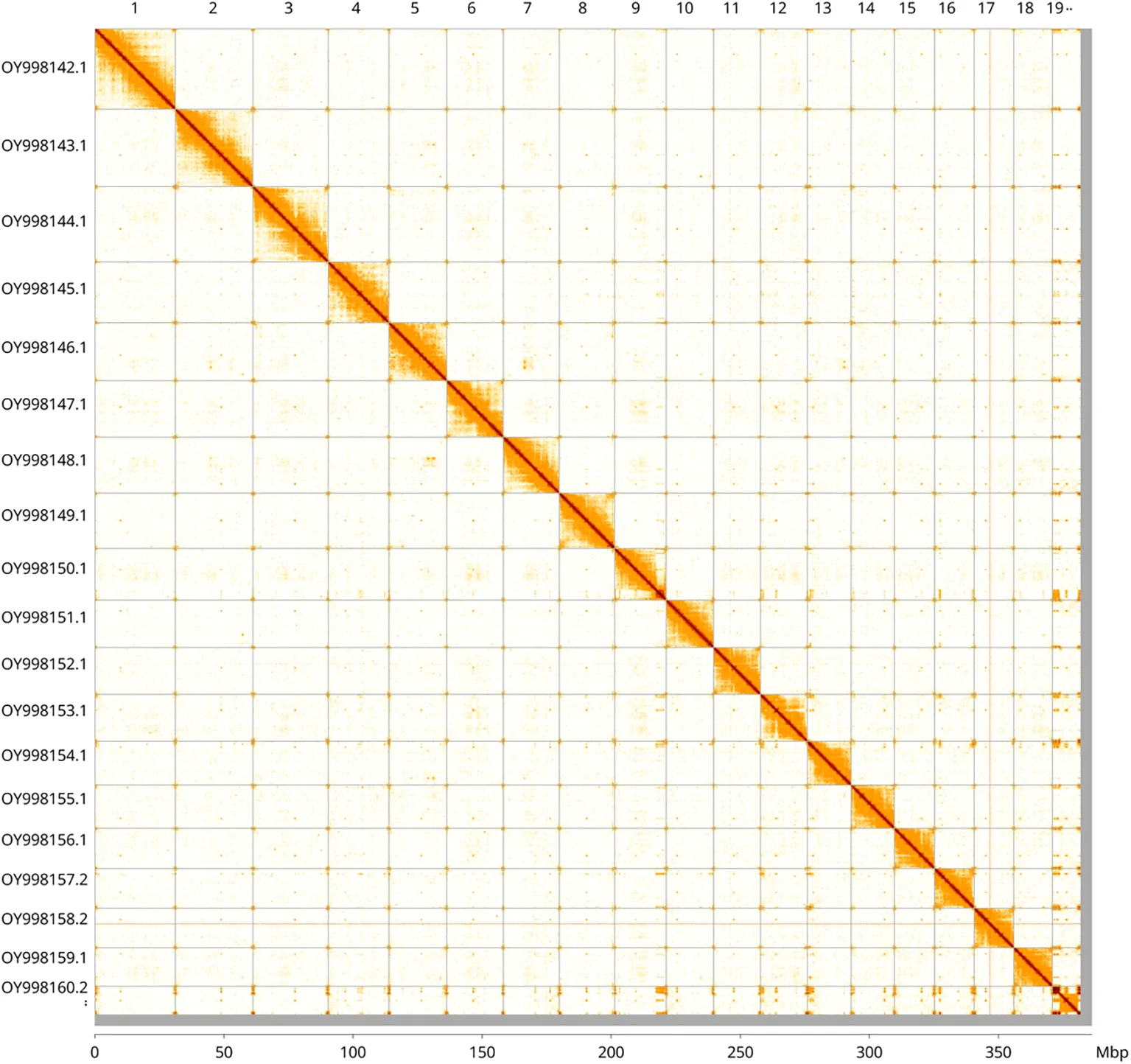
Hi-C contact map of the *Sphagnum tenellum* genome assembly. Assembled chromosomes are shown in order of size and labelled along the axes, with a megabase scale shown below. The plot was generated using PretextSnapshot.

Most of the assembly sequence (98.04%) was assigned to 21 chromosomal-level scaffolds. These chromosome-level scaffolds, confirmed by Hi-C data, are named according to size ( Figure 3; Table 3).

**Table 3.** Chromosomal pseudomolecules in the haploid genome assembly of *Sphagnum tenellum* csSphTene1.

INSDC accession	Molecule	Length (Mb)	GC%
OY998142.1	1	31.02	37
OY998143.1	2	29.71	36.50
OY998144.1	3	28.87	36.50
OY998145.1	4	23.28	37
OY998146.1	5	22.27	36.50
OY998147.1	6	21.67	36.50
OY998148.1	7	21.55	36.50
OY998149.1	8	21.22	37
OY998150.1	9	19.82	36.50
OY998151.1	10	18.18	36.50
OY998152.1	11	18	36.50
OY998153.1	12	17.98	37
OY998154.1	13	16.83	36.50
OY998155.1	14	16.54	37
OY998156.1	15	15.42	36.50
OY998157.2	16	15.27	36.50
OY998158.2	17	15.18	37
OY998159.1	18	14.88	37
OY998160.2	19	8.25	37.50
OZ251488.1	20	1.49	38
OZ251489.1	21	1.16	37.50

The mitochondrial genome (length 141.31 kb, OY998161.1) and plastid genome (length 140.16 kb, OY998162.1) were also assembled. These sequences are included as contigs in the multifasta file of the genome submission and as standalone records.

### Assembly quality metrics

**Figure 4.**
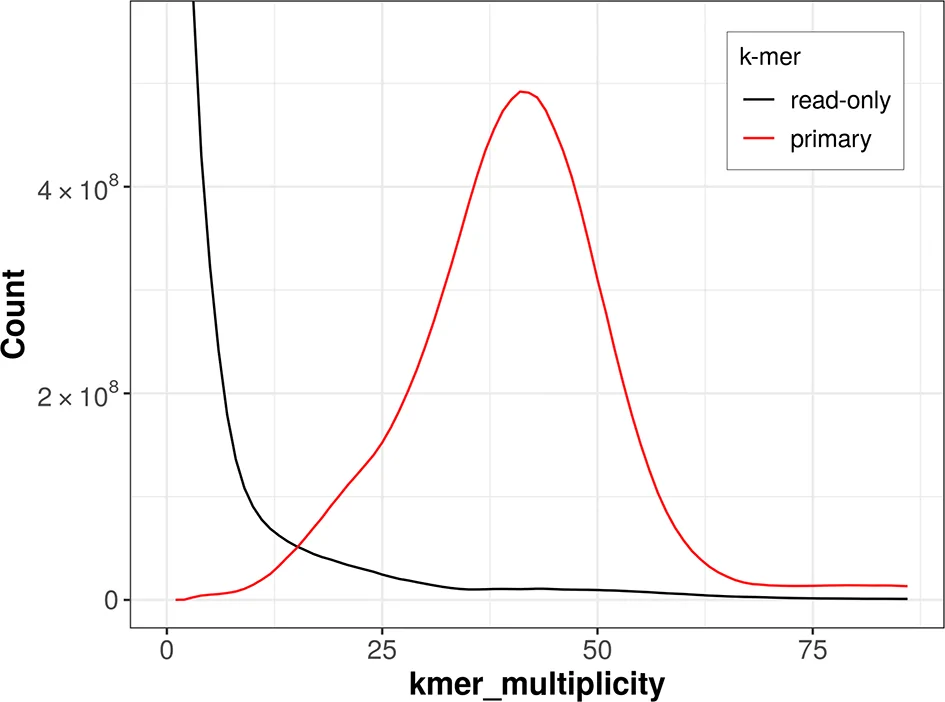
Evaluation of *k*-mer completeness using MerquryFK. This plot illustrates the recovery of *k*-mers from the original read data in the final haploid assembly. The horizontal axis represents *k*-mer multiplicity, and the vertical axis shows the number of *k*-mers. The black curve represents *k*-mers that appear in the reads but are not assembled, while the remaining curves correspond to *k*-mers recovered in the final assembly.

The haploid assembly achieves an estimated QV of 64.8. The *k*-mer completeness is 93.04% for the haploid assembly ( Figure 4).

**Figure 5.**
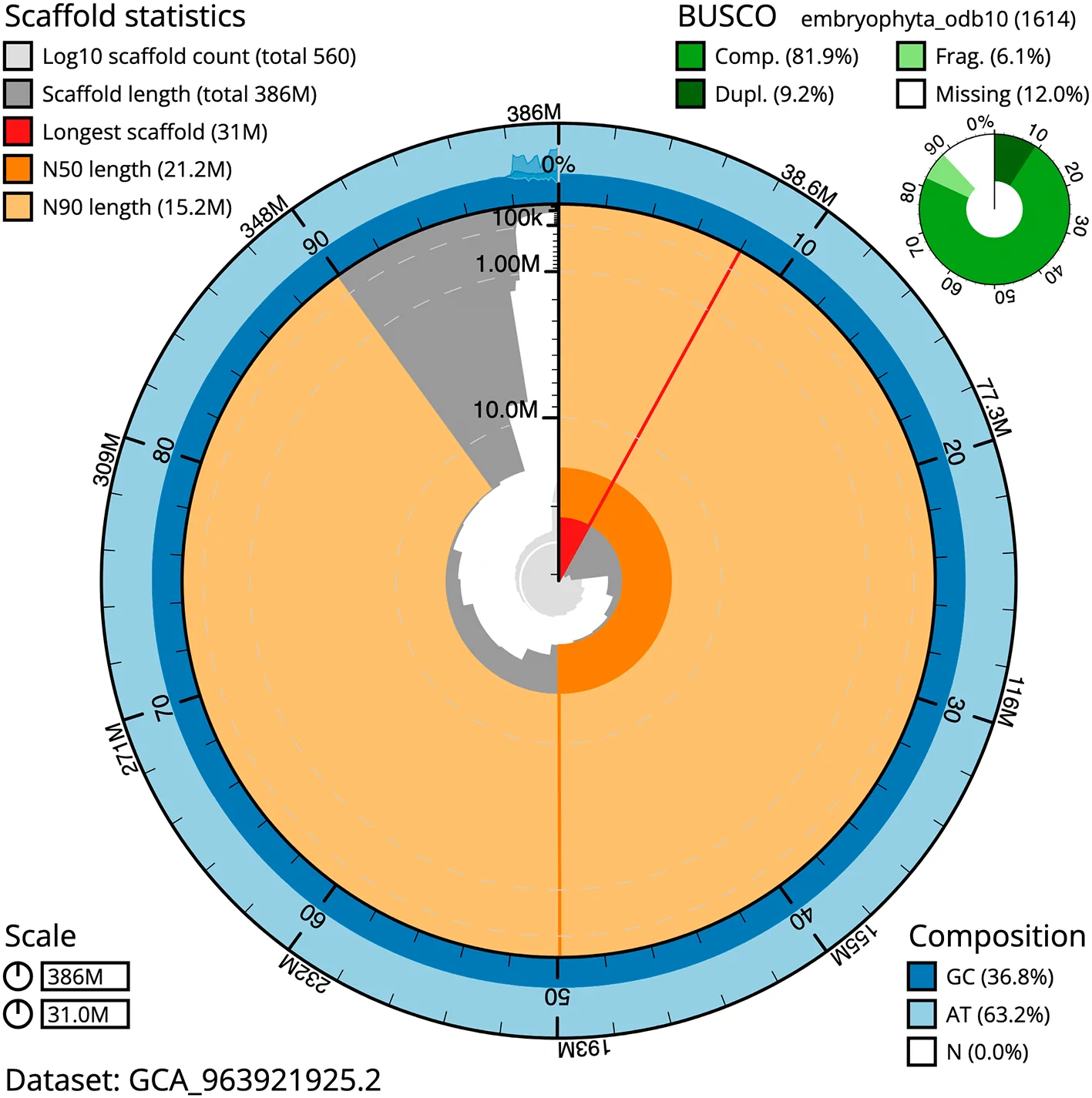
Assembly metrics for csSphTene1.2. The BlobToolKit snail plot provides an overview of assembly metrics and BUSCO gene completeness. The circumference represents the length of the whole genome sequence, and the main plot is divided into 1,000 bins around the circumference. The outermost blue tracks display the distribution of GC, AT, and N percentages across the bins. Scaffolds are arranged clockwise from longest to shortest and are depicted in dark grey. The longest scaffold is indicated by the red arc, and the deeper orange and pale orange arcs represent the N50 and N90 lengths. A light grey spiral at the centre shows the cumulative scaffold count on a logarithmic scale. A summary of complete, fragmented, duplicated, and missing BUSCO genes in the embryophyta_odb10 set is presented at the top right. An interactive version of this figure can be accessed on the BlobToolKit viewer.

**Figure 6.**
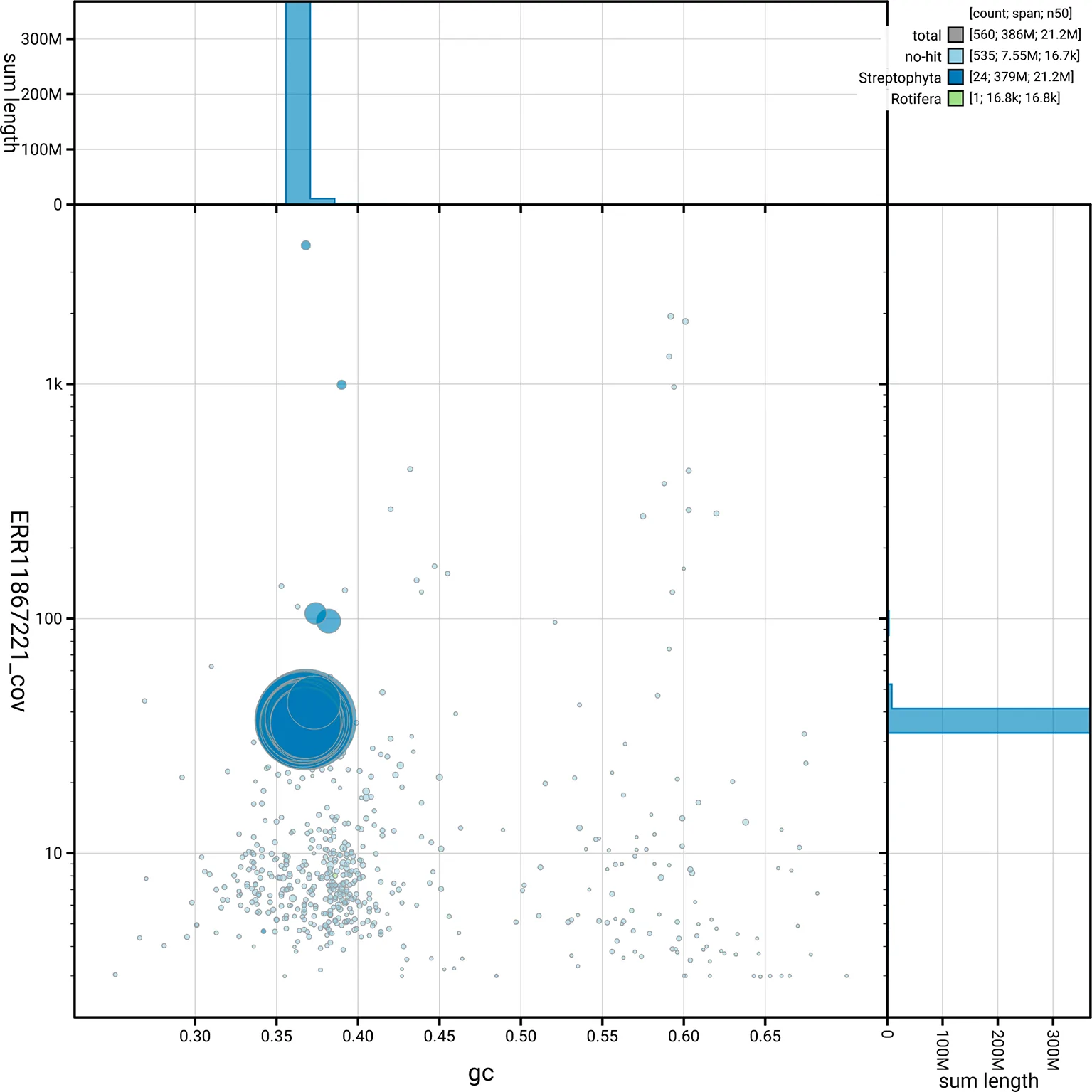
BlobToolKit blob plot for csSphTene1.2. The plot shows base coverage (vertical axis) and GC content (horizontal axis). The circles represent scaffolds, with the size proportional to scaffold length and the colour representing phylum membership. The histograms along the axes display the total length of sequences distributed across different levels of coverage and GC content. An interactive version of this figure is available on the BlobToolKit viewer.

BUSCO v.5.7.1 analysis using the embryophyta_odb10 reference set ( *n* = 1 614) identified 81.9% of the expected gene set (single = 72.7%, duplicated = 9.2%). The snail plot in Figure 5 summarises the scaffold length distribution and other assembly statistics for the haploid assembly. The blob plot in Figure 6 shows the distribution of scaffolds by GC proportion and coverage.

Table 4 lists the assembly metric benchmarks adapted from Rhie *et al.* (2021) and the Earth BioGenome Project Report on Assembly Standards September 2024. The EBP metric calculated for the haploid assembly is **6.C.Q64**, meeting the recommended reference standard.

**Table 4.** Earth Biogenome Project summary metrics for the *Sphagnum tenellum* assembly.

Measure	Value	Benchmark
EBP summary (haploid assembly)	6.C.Q64	6.C.Q40
Contig N50 length	1.03 Mb	≥ 1 Mb
Scaffold N50 length	21.22 Mb	= chromosome N50
Consensus quality (QV)	Haploid assembly: 64.8	≥ 40
*k*-mer completeness	Haploid assembly: 93.04%	≥ 95%
BUSCO	C:81.9% [S:72.7%, D:9.2%], F:6.1%, M:12.0%, n:1 614	S > 90%; D < 5%
Percentage of assembly assigned to chromosomes	98.04%	≥ 90%

*Notes:* The EBP summary uses log10(Contig N50); chromosome-level (C) or log10(Scaffold N50); Q (Merqury QV). BUSCO: C = complete; S = single-copy; D = duplicated; F = fragmented; M = missing; n = orthologues.

**Table 5.** Software versions and sources used for *Sphagnum tenellum.*

Software	Version	Source
BLAST	2.14.0	ftp://ftp.ncbi.nlm.nih.gov/blast/executables/blast+/
BlobToolKit	4.4.5	https://github.com/blobtoolkit/blobtoolkit
BUSCO	5.7.1	https://gitlab.com/ezlab/busco
bwa-mem2	2.2.1	https://github.com/bwa-mem2/bwa-mem2
DIAMOND	2.1.8	https://github.com/bbuchfink/diamond
fasta_windows	0.2.4	https://github.com/tolkit/fasta_windows
FastK	1.1	https://github.com/thegenemyers/FASTK
GenomeScope2.0	2.0.1	https://github.com/tbenavi1/genomescope2.0
Gfastats	1.3.6	https://github.com/vgl-hub/gfastats
Hifiasm	0.16.1-r375	https://github.com/chhylp123/hifiasm
HiGlass	1.13.4	https://github.com/higlass/higlass
MerquryFK	1.1.2	https://github.com/thegenemyers/MERQURY.FK
Minimap2	2.28-r1209	https://github.com/lh3/minimap2
MitoHiFi	3	https://github.com/marcelauliano/MitoHiFi
MultiQC	1.14; 1.17 and 1.18	https://github.com/MultiQC/MultiQC
Nextflow	24.10.4	https://github.com/nextflow-io/nextflow
PretextSnapshot	0.0.5	https://github.com/sanger-tol/PretextSnapshot
PretextView	1.0.3	https://github.com/sanger-tol/PretextView
purge_dups	1.2.5	https://github.com/dfguan/purge_dups
samtools	1.21	https://github.com/samtools/samtools
sanger-tol/ascc	0.1.0	https://github.com/sanger-tol/ascc
sanger-tol/blobtoolkit	v0.7.1	https://github.com/sanger-tol/blobtoolkit
sanger-tol/curationpretext	1.4.2	https://github.com/sanger-tol/curationpretext
Seqtk	1.3	https://github.com/lh3/seqtk
Singularity	3.9.0	https://github.com/sylabs/singularity
TreeVal	1.4.0	https://github.com/sanger-tol/treeval
YaHS	1.2a.2	https://github.com/c-zhou/yahs

## Author information

• Members of the Royal Botanic Garden Edinburgh Genome Acquisition Lab
• Members of the Plant Genome Sizing Collective
• Members of the Darwin Tree of Life Barcoding collective
• Members of the Wellcome Sanger Institute Tree of Life Management, Samples and Laboratory team
• Members of Wellcome Sanger Institute Scientific Operations – Sequencing Operations
• Members of the Wellcome Sanger Institute Tree of Life Core Informatics team
• Members of the Tree of Life Core Informatics collective
• Members of the Darwin Tree of Life Consortium

## Wellcome sanger institute – legal and governance

The materials that have contributed to this genome note have been supplied by a Darwin Tree of Life Partner. The submission of materials by a Darwin Tree of Life Partner is subject to the **‘Darwin Tree of Life Project Sampling Code of Practice’**, which can be found in full on the Darwin Tree of Life website. By agreeing with and signing up to the Sampling Code of Practice, the Darwin Tree of Life Partner agrees they will meet the legal and ethical requirements and standards set out within this document in respect of all samples acquired for, and supplied to, the Darwin Tree of Life Project. Further, the Wellcome Sanger Institute employs a process whereby due diligence is carried out proportionate to the nature of the materials themselves, and the circumstances under which they have been/are to be collected and provided for use. The purpose of this is to address and mitigate any potential legal and/or ethical implications of receipt and use of the materials as part of the research project, and to ensure that in doing so we align with best practice wherever possible. The overarching areas of consideration are:

• Ethical review of provenance and sourcing of the material
• Legality of collection, transfer and use (national and international)

Each transfer of samples is further undertaken according to a Research Collaboration Agreement or Material Transfer Agreement entered into by the Darwin Tree of Life Partner, Genome Research Limited (operating as the Wellcome Sanger Institute), and in some circumstances, other Darwin Tree of Life collaborators.

## Data Availability

European Nucleotide Archive: Sphagnum tenellum (soft bog-moss). Accession number PRJEB65221; https://identifiers.org/ena.embl/PRJEB65221. The genome sequence is released openly for reuse. The *Sphagnum tenellum* genome sequencing initiative is part of the Darwin Tree of Life Project (PRJEB40665) and the Sanger Institute Tree of Life Programme (PRJEB43745). All raw sequence data and the assembly have been deposited in INSDC databases. The genome will be annotated using available RNA-Seq data and presented through the Ensembl pipeline at the European Bioinformatics Institute. Raw data and assembly accession identifiers are reported in Tables 1 and 2. Pipelines used for genome assembly at the WSI Tree of Life are available at https://pipelines.tol.sanger.ac.uk/pipelines. Table 5 lists software versions used in this study.
