# Supplementary material for: The genome sequence of Sphagnum tenellum (Brid.) Bory, 1819 (Sphagnales: Sphagnaceae)
Source: Wellcome Open Res. 2026 May 29;11:316. [Version 1] doi: 10.12688/wellcomeopenres.26663.1 (PMC13555085; doi:10.12688/wellcomeopenres.26663.1)
Supplement: Peer review history

# Reviewer report: Reviewer response for version 1

**DOI:** 10.21956/wellcomeopenres.29363.r159785

**Published:** 2026-07-20

**Authors:** Elena Apostolova-Kuzova

**Affiliations:** 1 Paisii Hilendarski University of Plovdiv, Plovdiv, Bulgaria

The article entitled "The genome sequence of *Sphagnum tenellum* (Brid.) Bory, 1819 (Sphagnales: Sphagnaceae)" by David Bell and co-authors presents a high-quality genome assembly of *Sphagnum tenellum*. The majority of the nuclear genome assembly is scaffolded into 21 chromosomal pseudomolecules. In addition, the authors provide complete mitochondrial and plastid genome sequences. This high-quality reference genome represents a valuable genomic resource that will facilitate a wide range of future studies on this ecologically important bryophyte group. The material provided and details of the applaied methods are detiled presented and would allow replication by other scientists. The resulted sequences are annotated and available in the public databases Ensembl/NCBI.

 The authors cite the previously published chromosome-level assembly of *Sphagnum tenellum *but do not discuss why the current assembly contains 21 chromosome-scale pseudomolecules, whereas the previous assembly reported 20. Given that cytological studies consistently report a haploid chromosome number of n = 19 for this species, a brief discussion explaining whether this discrepancy reflects differences in scaffolding, assembly curation, or structural variation between accessions would considerably strengthen the manuscript.

Are sufficient details of methods and materials provided to allow replication by others?

Yes

Is the rationale for creating the dataset(s) clearly described?

Yes

Are the datasets clearly presented in a useable and accessible format?

Yes

Are the protocols appropriate and is the work technically sound?

Yes

Reviewer Expertise:

Molecular biology, Bioinformatic analyses

I confirm that I have read this submission and believe that I have an appropriate level of expertise to confirm that it is of an acceptable scientific standard.

# Reviewer report: Reviewer response for version 1

**DOI:** 10.21956/wellcomeopenres.29363.r159789

**Published:** 2026-07-10

**Authors:** John L. Bowman

**Affiliations:** 1 Monash University, Melbourne, Australia

The genome sequence of *Sphagnum tenellum *(Brid.) Bory, 1819

 Bell et al.

 The sequence presented appear to be of high quality and will be a resource for those studying *Sphagnum* genomics.

 A few specific comments.

 1. As *Sphagnum tenellum *is a dioicous species, it would be useful to know the sex or at least provide some specific information about the putative sex chromosome. By my reckoning, unfortunately, the other *Sphagnum tenellum *genome is also of the same sex as even the putative sex chromosomes are similar, which would not occur if they were of opposite sexes.  A few words about this would be useful.

 2. It is stated that the genome exhibits a heterozygosity of 0.61% — how was this determined? was it a comparison between csSphTene1 and csSphTene6? It seems a better comparison would be to determine the polymorphism frequency between this genome and the Elixir Norway assembly.

 3. While likely not a purview of this manuscript, several other *Sphagnum* genomes are available for comparison — is there anything striking about this genome or is it similar in most aspects to those already available?

Are sufficient details of methods and materials provided to allow replication by others?

Yes

Is the rationale for creating the dataset(s) clearly described?

Yes

Are the datasets clearly presented in a useable and accessible format?

Yes

Are the protocols appropriate and is the work technically sound?

Yes

Reviewer Expertise:

Developmental and evolutionary genetics

I confirm that I have read this submission and believe that I have an appropriate level of expertise to confirm that it is of an acceptable scientific standard, however I have significant reservations, as outlined above.

# Reviewer report: Reviewer response for version 1

**DOI:** 10.21956/wellcomeopenres.29363.r157169

**Published:** 2026-06-19

**Authors:** Anshul Dhyani

**Affiliations:** 1 Department of Botany, Hindu College, University of Delhi, New Delhi, Delhi, India; 2 University of Delhi, New Delhi, Delhi, India

I have gone through the manuscript entitled ‘ *The genome sequence of Sphagnum tenellum (Brid.) Bory, 1819 (Sphagnales: Sphagnaceae)*’. The manuscript is well-written and can be very useful in studying genome sequence and assembly of *Sphagnum*. However, I have a few comments which need justification before accepting the final version. Please find below the comments needed to be addressed by the authors:

1. The authors should add a paragraph in the Genome sequence report section addressing the BUSCO result explicitly. This should include: (a) a comparison with BUSCO completeness values from other published *Sphagnum* assemblies if available; (b) a discussion of whether the embryophyta_odb10 reference set is well-suited to Sphagnopsida, which diverged early from the land plant lineage; and (c) a brief explanation of the 9.2% duplication rate.
2. Add a comparison table or paragraph contrasting the key assembly metrics of GCA_963921925.2 (this study) and GCA_965154135.1 (Elixir Norway), including at minimum: total assembly length, number of chromosomes, scaffold N50, contig N50, QV, k-mer completeness, and BUSCO completeness. If one assembly is demonstrably superior in specific metrics, this should be stated directly. If the assemblies are complementary (e.g., from different individuals or sequencing strategies), this should be made explicit.
3. Add at minimum two to three sentences in the Genome sequence report section describing: (a) whether the organelle assemblies are circular or linear; (b) an estimated gene count for each; and (c) a brief comment on whether the mitochondrial genome size is consistent with other *Sphagnum* species. If the mitochondrial assembly is notably compact for a land plant, this warrants explicit discussion as it may be biologically informative or reflect assembly limitations.
4. Provide a brief explanation for the 5.8% reduction in assembly length during curation. Specifically, state whether this resulted from removal of contaminant sequences (and if so, which taxa), resolution of haplotypic duplications, or other factors. A single sentence of clarification would be sufficient.
5. Table 3 shows that chromosomes 1–19 range from 8.25 to 31.02 Mb, while chromosomes 20 and 21 are drastically smaller at 1.49 Mb and 1.16 Mb respectively, and carry slightly elevated GC content (38% and 37.5% vs the 36.5–37% range of the other chromosomes). These two chromosomes are likely sex chromosomes or micro chromosomes, but this is not discussed anywhere in the manuscript.

Are sufficient details of methods and materials provided to allow replication by others?

Yes

Is the rationale for creating the dataset(s) clearly described?

Yes

Are the datasets clearly presented in a useable and accessible format?

Partly

Are the protocols appropriate and is the work technically sound?

Yes

Reviewer Expertise:

Bryophyte Taxonomy, Phylogeny, DNA Barcoding, Cytogenetics of lower and higher plants

I confirm that I have read this submission and believe that I have an appropriate level of expertise to confirm that it is of an acceptable scientific standard, however I have significant reservations, as outlined above.
